# Endoscopic Treatment of Vesicoureteral Reflux with Dextranomer/Hyaluronic Acid in Children

**DOI:** 10.1155/2008/513854

**Published:** 2008-06-05

**Authors:** Wolfgang H. Cerwinka, Hal C. Scherz, Andrew J. Kirsch

**Affiliations:** Children's Healthcare of Atlanta, Emory University School of Medicine, 5445 Meridian Mark Road, Suite 420, Atlanta, GA 30342, USA

## Abstract

*Purpose*. The goal of this review is to present current indications, injectable agents, techniques, success rates, complications, and potential future applications of endoscopic treatment for vesicoureteral reflux (VUR) in children. *Materials and Methods*. The endoscopic method currently achieving one of the highest success rates is the double hydrodistention-implantation technique (HIT). This method employs dextranomer/hyaluronic acid copolymer, which has been used in pediatric urology for over 10 years and may be at present the first choice injectable agent due to its safety and efficacy. *Results*. While most contemporary series report cure rates of greater than 85% for primary VUR, success rates of complicated cases of VUR may be, depending on the case, significantly lower. Endoscopic treatment offers major advantages to patients while avoiding potentially complicated open surgery. As the HIT method continues to be applied to complex cases of VUR and more outcome data become available, the indication for endoscopic treatment may exceed the scope of primary VUR. *Conclusions*. Endoscopic injection is emerging as the treatment of choice for VUR in children.

## 1. INTRODUCTION

**Figure 1:**
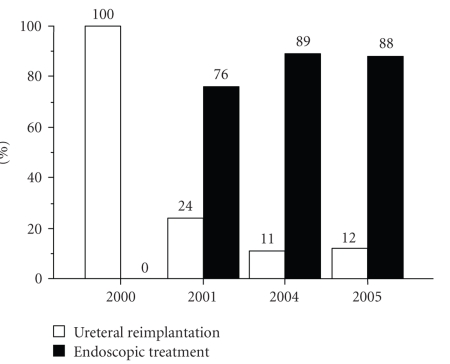
Trend of surgical treatment for VUR at Children’s Healthcare of Atlanta from 2000 to 2005.

Vesicoureteral reflux (VUR) is diagnosed in approximately 1% of children and promotes pyelonephritis, which may lead to renal scarring and hypertension [1]. VUR is one of many treatable risk factors (e.g., dysfunctional elimination) in the development of urinary tract infection (UTI). Treatment intends to prevent pyelonephritis and to preserve renal function and most children diagnosed with VUR receive antibiotic prophylaxis regardless of VUR grade [2]. Surgical management is indicated in cases of break-through UTIs and/or persistence of VUR and comprises ureteral reimplantation and endoscopic injection. Since the introduction of endoscopic treatment for VUR in 1981 and its first clinical application in 1984 as subureteric Teflon injection (STING), injection techniques, injectable agents, and consequently treatment success rates have considerably improved [3–6]. Endoscopic treatment not only approaches success rates of open ureteral reimplantation but offers also significant advantages to patients and parents such as outpatient surgery, lower morbidity (e.g., pain, scar), fewer complications, and reduced cost. Consequently, a major shift from reimplantations toward injection treatments has been observed over the last few years (Figure 1).

The purpose of this review is to summarize current indications, injectable agents, techniques, success rates, complications, and potential future applications of endoscopic treatment for VUR.

## 2. MATERIALS AND METHODS

### 2.1. Goals of treatment

There is little evidence that antireflux surgery of any means decreases the incidence of renal scarring or end-stage renal disease. Worthwhile goals of treatment are to prevent UTIs, particularly febrile UTIs, to avoid long-term antibiotic use, and to lessen the need for distressing voiding cystourethrographies (VCUG) and radiation exposure. Proponents of the endoscopic approach will argue that decreasing the incidence of UTI is the main goal of therapy. Recurrence, while possible, may occur in the absence of symptoms and be viewed as subclinical, similar to an individual with VUR diagnosed after a sibling screen or for fetal hydronephrosis. Proponents of the open surgical approach will argue that ureteral reimplantation provides a permanent cure of VUR and is worth the increased morbidity to achieve this goal. In terms of reducing the risk of UTI, endoscopic treatment may achieve this goal as well or better than open surgery [7–9].

### 2.2. Indications

The indications for ureteral reimplantation and endoscopic treatment are with few exceptions identical and comprise recurrent UTIs despite antibiotic prophylaxis, persistent VUR after a period of observation (>2 years), poor compliance with antibiotic prophylaxis, and new renal scarring. When parents are counseled regarding surgical options, a significant preference for endoscopic treatment is apparent [10, 11]. Endoscopic injection has more frequently been employed for primary rather than for complex VUR (i.e., VUR associated with functional or anatomical abnormalities such as neurogenic bladder or ectopic or megaureters). Avoidance of endoscopic treatment for complex VUR is due to a paucity of supportive clinical data and the current view that bladder dysfunction and structural defects of the ureterovesical junction necessitate ureteral reimplantation. Endoscopic treatment is Food and Drug Administration (FDA)-approved for VUR grades II to IV and for cases of initial endoscopic treatment failure, however it has been applied to all VUR scenarios. While open ureteral reimplantation is the treatment of choice for failed injection therapy, endoscopic treatment has been successfully employed after failed ureteral reimplantation [12–14]. In general, endoscopic treatment is emerging as the treatment modality of choice for VUR whereas ureteral reimplantation remains reserved for cases of failed injection therapy, significant anatomical abnormalities (e.g., large paraureteral diverticula, ectopic ureters, megaureters), and surgeon’s or parents’ preference.

### 2.3. Injectable agents

Numerous injectable bulking materials have been utilized and abandoned over time in search for the ideal agent, which should be nonimmunogenic, noncarcinogenic, biocompatible, and biodegradable. Teflon, the first bulking material used for the treatment of VUR, was abandoned in pediatric urology in the USA because of the material’s propensity to migrate to distant organs and to form granulomas; however, carcinogenesis of Teflon has not been reported [15–17]. Silicone also demonstrates distant migration and granuloma formation. Its carcinogenic potential has been controversial but is most likely unsubstantiated [18, 19]. Glutaraldehyde cross-linked bovine collagen demonstrates a lower degree of absorption as compared to native collagen and can cause allergic reactions even in patients with negative skin test [20]. Several new bulking agents are currently under investigation, such as inorganic materials and autologous tissue. The latter is nonimmunogenic, however, cell harvest and/or cell culture are time-consuming and expensive. Dextranomer/hyaluronic acid copolymer (Deflux, Q-Med Scandinavia Inc., Uppsala, Sweden) is easy to inject, is biodegradable with stable implant volume, and its relatively large particle size prevents distant migration [21, 22]. It has been used as injectable material in pediatric urology for over 10 years and is currently the first-choice injectable agent due to its safety and efficacy. Deflux implants in animal tissue were shown to undergo time-dependent histopathological changes. The initial phase was dominated by an ingrowth of granulation tissue, a foreign-body giant-cell reaction, and the formation of a surrounding capsule. In the later phase, cellular elements were largely replaced by a collagen-rich matrix, whereas the capsule remained unchanged [21]. These findings were confirmed in patients who experienced failed endoscopic injection and proceeded to ureteral reimplantation [22]. In our experience, explanted Deflux appears essentially unchanged up to 4 years after implantation. Besides biological properties, cost of bulking agents, and surgeon’s experience, the choice may ultimately depend on approval by administrative agencies, such as the European Medicines Agency or the FDA.

### 2.4. Technique

**Figure 2:**
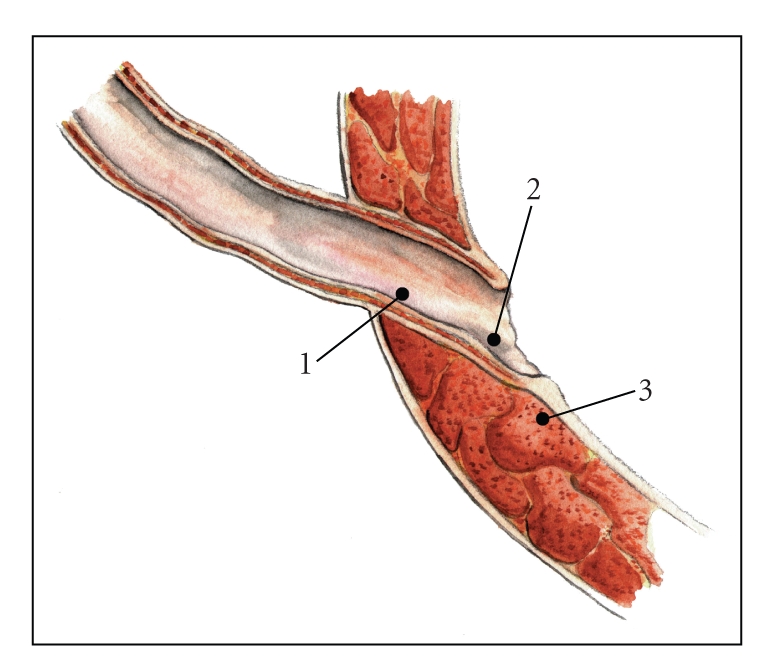
Needle placement algorithm for the endoscopic treatment of VUR. Sites 1 and 2 comprise the double-HIT method, while site 3 (STING) is rarely used.

The endoscopic method currently achieving one of the highest success rates is the double hydrodistention-implantation technique (HIT). Cystoscopy is performed with a pediatric cystoscope equipped with an off-set lens. An off-set lens permits direct passage of the needle in line with the ureter without bending the needle. The bladder is filled to less than half capacity to permit visualization of the ureter and avoid tension within the submucosal layer of the ureter secondary to overdistention. Hydrodistention (HD) is performed with the tip of the cystoscope placed at the ureteral orifice (UO), a pressured stream achieved by placing the irrigation bag approximately 1 meter above the bladder on full flow. HD of the distal ureter serves two purposes: it allows visualization of the intraureteral injection site and assessment of treatment progress (i.e., ureteral coaptation). The needle is passed into the UO and inserted at the mid ureteral tunnel at the 6 o’clock position. Sufficient bulking agent is injected to produce a bulge, which initially coapts the detrusor tunnel, while a second implant within the most distal intramural tunnel leads to coaptation of the UO (approximately 1–1.5 mL). Rarely, if the two intraureteric submucosal injections (double-HIT method) fail to coapt the ureter, a classic STING or a supraureteric injection is needed to achieve coaptation. The latter two injection sites are used more commonly in complex or redo cases (Figure 2). HD is performed after each injection to monitor treatment progress; when HD ceases to dilate the UO, appropriate coaptation has been achieved. At our institution, all procedures are performed on an outpatient basis and all patients receive preoperative antibiotic prophylaxis, which is continued until resolution of VUR has been confirmed. Radiographic success is defined as grade 0 VUR on a postoperative VCUG, from 1 to 3 months after a single treatment. Patients are then followed clinically on an annual basis to determine clinical success and recurrence.

We have shown that the Deflux bleb size, determined by ultrasound, correlates with treatment success; a measured volume higher than 25% of the injected volume using the HIT method will result in a 90% cure and 95% using the double HIT method [23, 24]. Consequently, as part of a prospective clinical trial we are evaluating bladder ultrasounds from 4 to 6 weeks after endoscopic injection with measurement of the Deflux bleb. If the retained volume is >25%, antibiotics will be discontinued, no VCUG will be obtained until 1 year, and the patient will be followed clinically. An earlier VCUG will be obtained for volumes <25% or if clinically indicated. This protocol allows for the identification of clinically significant VUR and will evaluate the longer-term success rate. If the long-term VCUGs show favorable results and/or if patients do well clinically, the postoperative VCUG will be excluded in the future.

## 3. RESULTS AND DISCUSSION

### 3.1. Success rates

Outcome of endoscopic treatment for VUR has been evaluated in several large series (Table 1). Most studies included both, primary and complicated cases of VUR. Interpretation of and comparison among these studies are confounded by different inclusion criteria (e.g., with or without complex VUR, grade I, grade V), varying lengths of follow-up, definitions of success, and single versus multiple injections. Nevertheless, most current series report cure rates of greater than 85%. Age, gender, and bilaterality of VUR have not been shown to predict treatment outcome. While the STING technique yields lower success rates with higher grades of VUR, the HIT method achieves similar outcomes across all VUR grades up to grade V [5]. Endoscopic treatment of complicated VUR has been evaluated in smaller series and success rates vary significantly depending on the associated pathologies (Table 2). In general, cure rates for complex cases of VUR are lower than for primary VUR. Treatment of VUR associated with neurogenic bladder was shown to yield acceptable outcome whereas voiding dysfunction was a significant predictor of treatment failure [13, 25]. Endoscopic injection has been successfully employed in patients who either failed ureteral reimplantation or initial injection [13, 14, 26]. Injection after failed reimplantion or second injection will be curative in most instances whereas a third injection has been shown to be far less successful [27, 28]. Refluxing ureters of transplanted kidneys in symptomatic patients may be treated endoscopically. Although this approach is curative in only half the cases, yet it represents an attractive alternative to open surgery in the setting of immune compromise and reduced wound healing properties [29]. VUR associated with anatomical abnormalities, previously thought to be contraindications for endoscopic treatment, was recently shown to be amenable to injection treatment [30–32].

There are many factors that may affect the success of the procedure. Preoperative (i.e., patient selection), intraoperative (i.e., injection technique), and postoperative variables have been shown to correlate with treatment outcome (Table 3). Postoperatively, failures may result from Deflux displacement (implant migration), disruption (mucosal breach), or dissolution (decrease in implant volume).

### 3.2. Advantages

In comparison to ureteral reimplantation, endoscopic VUR treatment offers major advantages to patients and parents. The procedure generally lasts less than 15 minutes and is performed on an outpatient basis. While cure rates are approaching those of open ureteral reimplantation, significant complications are rare. Endoscopic treatment entails greater patient convenience, lower morbidity (e.g., pain, abdominal scar), and reduced cost [43, 44]. Consequently, a significant parental preference for endoscopic treatment is evident [10, 11]. A recent study demonstrated that both, patients and parents viewed injection therapy as the least bothersome aspect of VUR treatment followed by antibiotic prophylaxis and VCUG [8].

### 3.3. Complications

The most common complications following endoscopic treatment of VUR are new contralateral VUR (2.3–17.3%) and treatment failure [35, 38]. Less than 4% of children complain of flank pain or emesis several hours after the procedure and all respond to analgesics [5]. Gross hematuria, urinary retention, or febrile UTIs have not been observed. The most significant potential complication of endoscopic treatment for VUR includes a 0.6% risk of ureteral obstruction [45]. Our obstruction rate is 4 ureters (2 patients) in over 1200 ureteral injections, or <0.3%. A 7-month old boy with bilateral grade V VUR and spina bifida developed acute renal failure and had bilateral ureteral stents placed. A STING technique using 0.7 mL was utilized. A postoperative VCUG after stent removal showed bilateral grade V VUR and a vesicostomy was performed. Interestingly, no VUR was seen at the time of bladder augmentation 5 years later. A 6-year old girl developed bilateral ureteral obstruction after HIT using 1.1 mL and 0.7 mL. Bilateral nephrostomy tubes were placed and removed 6 weeks later after a normal antegrade study. The VCUG did not show any evidence of VUR and no further treatment was required.

Factors that may increase the risk of obstruction include bladder dysfunction and markedly dilated ureters. Patients with recurrent VUR often remain asymptomatic and without risk factors for pyelonephritis such as young age, voiding dysfunction, or significant history of UTIs may be taken off antibiotic prophylaxis [46]. Febrile UTIs after radiographically successful endoscopic treatment warrant evaluation for recurrent VUR.

### 3.4. Potential future applications

As endoscopic treatment continues to be applied to more complex cases of VUR and outcome data become available, the indication for endoscopic treatment may exceed the scope of primary VUR. In the USA, for example, duplex ureters are no longer considered a contraindication for endoscopic treatment with Deflux by the FDA. Outcome analysis of complex cases of VUR will aid in preoperative counseling and patient selection and paired with proper technique further improve success rates of endoscopic VUR treatment. As adults with recurrent pyelonephritis are more consistently evaluated for VUR, a patient population with distinct requirements and disease characteristics may emerge. Finally, once more accurate predictors of VUR resolution/persistence become available, endoscopic treatment may be more frequently used as the primary treatment in patients with low probability of VUR resolution.

## 4. CONCLUSIONS

Endoscopic treatment of VUR offers significant advantages to patients and avoids potentially complicated open surgery. While success of endoscopic treatment for primary VUR approaches that of ureteral reimplantation, it is acceptable in complex cases of VUR. Consequently, endoscopic injection has assumed the role of first-line VUR treatment whereas ureteral reimplantation remains reserved for cases of failed injection therapy or significant anatomical abnormalities. The development of new injectable agents in combination with the improvement of endoscopic techniques will continue to strengthen the role of endoscopic treatment for VUR.

## Figures and Tables

**Table 1:** Success rates of endoscopic treatment for primary VUR. Meta-analysis by Elder JS et al. 2006 summarizes results until 2003. More recent series are listed below. Initial success after one treatment and final success after two or more treatments.

Reference	Bulking agent	Injected volume	Ureters	Follow-up	Success	
					Initial	Final
Elder et al. 2006 [27]	Various	0.2–1.7 mL	8101	variable	76%	85%
Capozza et al. 2004 [33]	Various	0.2–2.2 mL	1694	12–204 months		77%
Kirsch et al. 2004 [5]	Dx/HA	0.5–1.5 mL	119	3–12 months	92%	
Kirsch et al. 2006 [34]	Dx/HA	0.8–2.0 mL	139	3–18 months	93%	
Van Capelle et al. 2004 [35]	PDMS	0.2–2 mL	311	3–110 months		75%
Kajbafzadeh et al. 2006 [36]	Ca hydroxylapatite	0.4–0.6 mL	364	6 months		69%
Yu and Roth 2006 [6]	Dx/HA	1 mL	162	2–26 months	87%	93%
Puri et al. 2006 [37]	Dx/HA	0.2–1.5 mL	1101	3–46 months	87%	96%
Lorenzo et al. 2006 [38]	PDMS		351	72 months		72%
Pinto et al. 2006 [39]	Dx/HA		86	3 months		84%

**Table 2:** Success rates of endoscopic treatment for complex VUR.

Reference	Pathology	Bulking agent	Injected volume	Ureters	Follow-Up	Success
Perez-Brayfield et al. 2004 [13]	Neurogenic bladder	Dx/HA	0.4–2.0 (1.1)	9	3 months	78%
Capozza et al. 2002 [25]	Voiding dysfunction	Dx/HA			3–6 months	49%
Elmore et al. 2006 [26]	Failed initial injection	Dx/HA	1.0–1.5	53	3 months	89%
Perez-Brayfield et al. 2004 [13]	Failed reimplantation	Dx/HA	0.4–2.0 (1.1)	19	3 months	88%
Kitchens et al. 2006 [14]	Failed reimplantation	Dx/HA	0.7–3.8 (0.8)	20	19 months	83%
Campbell et al. 2006 [29]	Renal transplantation	Dx/HA		11		55%
Molitierno et al. 2007 [30]	Duplicated ureter	Dx/HA	0.8–2.8 (1.4)	63	1–3 months	85%
Cerwinka et al. 2007 [31]	Paraureteral diverticulum	Dx/HA	0.8–1.8 (1.2)	20	6.6 months	81%
Chertin et al. 2007 [32]	Ureterocele	Various		44	1–21 months	91%

**Table 3:** Variables affecting the outcome of endoscopic treatment of VUR with Deflux. Overall success for patients/ureters.

Reference	Bulking agent	Patients/Ureters	Mean age	Overall success	Predictors of success	Not predictive
Lavelle et al. 2005 [40]	Dx/HA	52/80	7.6 years	71%/80%	Volcano: present 87%	Voiding dysfunction
					* * * * * *absent 53%	VUR grade
						Injected volume
Yucel et al. 2007 [41]	Dx/HA	168/259	4.2 years	82%/86%	Volcano: present 87%	Voiding dysfunction
					* * * * * *absent 36%	Laterality
					Volume: <0.5 mL success	
					>0.5 mL failure	
					VUR grade	
Routh et al. 2007 [42]	Dx/HA	301/453	5.5 years	66%/72%	VUR grade	
					Surgeon	

## ABBREVIATIONS

VUR: Vesicoureteral reflux
UTI: Urinary tract infection
STING: Subureteric Teflon injection
VCUG: Voiding cystourethrography
FDA: Food and Drug Administration
HIT: Hydrodistention Implantation Technique
HD: Hydrodistention
UO: Ureteral orifice.
